# A Case of Retained Placenta

**Published:** 1843-10-14

**Authors:** 


### A CASE OF RETAINED PLACENTA.

To the Editor of the Medical Examiner.

Sir:—On Sunday, July 23d, 1843, at 11 o'clock A. M. I was called to visit Mrs. M. S. On arriving in her chamber I learned that a miscarriage had just taken place, and a fœtus of about three or four months was shown to me. Upon inquiring into Mrs. S.'s history, I gathered the following particulars:—that she was afflicted with habitual abortion, this being the *fourteenth* child which she had aborted: that in this, as well as in every previous case there were *no premonitory symptoms*, the expulsion of the child being almost the first indication of the accident: that she never suffered from uterine or labour pains, even when she carried her child until the full period, which she had done in two instances: that invariably the expulsion of the child was followed by profuse hæmorrhage, which continued in spite of every treatment several days, exhausting her strength to such a degree that it required months to regain it: and lastly, that the placenta never came away without assistance.

Upon making an examination I found, as I had been informed, that my patient was flooding profusely. The uterus was low down in the pelvic cavity, and the os uteri was dilated about an inch. I readily introduced my index finger, and guided by the umbilical cord, I succeeded in finding the placenta. It was firmly attached to the left side of the womb. With some difficuly I detached and brought away about one third of the placental mass, but the hæmorrhage being thereby increased to an alarming extent, I deemed it best to desist from using any further manual means.

I now introduced *the tampon*, had ice-water applications made to the lower part of the abdomen, and directed ten grains of the ergot of rye to be administered every half hour until I should call again. It was about 12 o'clock. At 2, P. M., I again visited my patient, and on entering the room Mrs. S. exclaimed, "Oh, Doctor I cannot tell what is about to befall me! Soon after you left me I was taken with *severe bearing down pains*, and they have continued with but little intermission until now;—such pains I never experienced before." Knowing that the ergot had brought on uterine contractions, I told her that every thing was just as I had intended. The tampon still retained its place in the vagina, and there was an external appearance of hæmorrhage. The patient felt stronger and had revived considerably since the morning. Two more of the ergot powders remaining, I directed them to be taken at one hour interval.

10 o'clock, P. M. I made my third visit. I found, to my great satisfaction, that during the evening the tampon along with the remnant of the placenta had been expelled, and that the flooding had entirely ceased.

Mrs. S. continued to improve daily, and on the 27th inst., four days after the miscarriage, I found her stirring about her domestic affairs.

Guided by the history which I obtained from the patient, I can readily account for the flooding from which she invariably suffered, and for the fact that the placenta never came away unassisted. She "never knew what it was to have a labour pain," we may consequently infer that there was a want of the usual tonic contractions of the uterus. And to this circumstance may both the flooding and the difficulty of voiding the after-birth be referred, as the uterus did not sufficiently contract to arrest the one or expel the other.

The indication in the case was plain:—to bring on uterine contractions. This, as I have related, was accomplished by the specific action of ergot: and by its use along with the tampon, the female was saved the suffering which would have resulted from removing the placenta mechanically, as well as the exhaustion which in every previous case resulted from the long continued flooding.

Mrs. S. is but recently from the State of New Jersey. She states that not unfrequently she has been under the Doctor's hands for two hours before the placenta could be removed, and in one instance, to use her own words, "it was forcibly dug away by the handle of a spoon."

A.

*Philadelphia*, Sept. 23, 1843.

